# Pancreatic Cancer Detection in Intraductal Papillary Mucinous Neoplasm (IPMN)—New Insights

**DOI:** 10.3390/cancers17203341

**Published:** 2025-10-16

**Authors:** Wojciech Pawłowski, Mateusz Stefański, Barbara Włodarczyk, Łukasz Durko, Ewa Małecka-Wojciesko

**Affiliations:** Department of Digestive Tract Diseases, Medical University of Lodz, 90-153 Lodz, Poland; barbara.wlodarczyk@umed.lodz.pl (B.W.); lukasz.durko@umed.lodz.pl (Ł.D.); ewa.malecka-panas@umed.lodz.pl (E.M.-W.)

**Keywords:** IPMN, pancreatic cancer, pancreatic cyst, pancreatic cystic neoplasm, radiomics, MRI, CT, EUS

## Abstract

**Simple Summary:**

Intraductal papillary mucinous neoplasm is a precancerous form of pancreatic cancer and has posed a diagnostic challenge for years. In recent years, new guidelines, especially those from the International Association of Pancreatology, have been developed to systematize malignancy risk factors and propose management strategies. New techniques, particularly those based on artificial intelligence and radiomics, have significantly improved the diagnostic accuracy of magnetic resonance imaging and computed tomography, which remain the most commonly used modalities for assessing intraductal papillary mucinous neoplasm. New molecular and endoscopic techniques, such as pancreatoscopy and endoscopic ultrasound–confocal laser endomicroscopy, are also rapidly advancing. This paper aims to summarize the current state of knowledge in diagnosing malignancy in intraductal papillary mucinous neoplasm, with particular emphasis on the latest diagnostic advances, in order to serve as a valuable clinical resource for practicing physicians.

**Abstract:**

Early diagnosis of pancreatic cancer, particularly in intraductal papillary mucinous neoplasm (IPMN), remains challenging despite advances in imaging and biomarkers. Pancreatic adenocarcinoma (PDAC) has a high mortality rate; therefore, its early detection and adequate interventions are necessary to improve the disease outcome. Most IPMNs are asymptomatic and discovered incidentally. Magnetic resonance imaging (MRI) is a preferred tool for diagnosing malignant IPMNs, with a sensitivity of 90.7–94.1% and a specificity of 84.7–87.2% in detecting mural nodules > 5 mm, a strong predictor of high-risk lesions. Radiomics further enhances diagnostic accuracy (sensitivity 91–96%, specificity 78–81%), especially when combined with CA 19-9, which has lower sensitivity (73–90%) but higher specificity (79–95%). Computed tomography (CT), though less effective for small mural nodules, remains widely used; its accuracy improves with radiomics and clinical variables (sensitivity 90.4%, specificity 74%). Conventional endoscopic ultrasonography (EUS) shows lower performance (sensitivity 60%, specificity 80%), but its advanced variations have improved outcomes. Contrast-enhanced EUS (CE-EUS) visualizes mural nodules with more than 90% sensitivity and involvement of the main pancreatic duct, with a sensitivity of 83.5% and a specificity of 87%. EUS–fine-needle aspiration (EUS-FNA) allows cyst fluid analysis; however, CEA, glucose, and KRAS/GNAS mutations show poor value for malignancy risk. Cytology has low sensitivity (28.7–64.8%) but high specificity (84–94%) in diagnostic malignant changes and strongly affects further management. EUS–through-the-needle biopsy (EUS-TTNB) yields high diagnostic accuracy (sensitivity 90%, specificity 95%) but carries a range of 2–23% adverse events, which limits its wide use. EUS–confocal laser endomicroscopy (EUS-nCLE) provides real-time microscopic evaluation, detecting malignant IPMN with a sensitivity of 90% and a specificity of 73%, though its availability is limited. New emerging biomarkers available in cyst fluid or blood include mucins, miRNA panels (sensitivity 66.7–89%, specificity 89.7–100%), lipidomics, and cancer metabolite profiling, with diagnostic accuracy approaching 89–91%. Pancreatoscopy (POP) enables direct main pancreatic duct (MPD) visualization and biopsy with a sensitivity of 64–100% and a specificity of 75–100%, though adverse events occur in around 12% cases. Combining advanced imaging, EUS-based tissue acquisition, and novel biomarkers holds promise for earlier and more accurate detection of malignant IPMN, potentially improving PDAC outcomes.

## 1. Introduction

Pancreatic ductal adenocarcinoma (PDAC) is one of the most fatal cancers. In 2022, 510,566 cases of PDAC were diagnosed worldwide, among which 467,005 died from this cause [1]. The incidence of PDAC is systematically increasing, and it mostly occurs in developed countries: the USA, Europe, Australia, and Argentina. PDAC is already the third-leading cause of cancer death in both sexes combined in the United States [2,3]. Unfortunately, PDAC remains asymptomatic for a long time, and most patients are diagnosed at a late stage when the disease is advanced and the tumor is unresectable [4]. The 5-year survival rate is only about 12%. In unresectable or metastatic disease, the 5-year survival rate is less than 3% [5,6].

Intraductal papillary mucinous neoplasm (IPMN) is one of the PDAC precursors [5]. IPMNs arise from the epithelial lining of the main pancreatic duct or/and its branches [6]. Pancreatic cysts, the most common feature of IPMNs [7,8], are increasingly detected due to the widespread use of high-quality imaging techniques [5,7,8,9]. It is estimated that 4–14% of the general population may have pancreatic cysts of a different nature [10]. IPMNs are estimated to be the most common type of cyst among all pancreatic cysts [7]. Morphologically, IPMN is divided into three subtypes, namely, main duct IPMN (MD-IPMN), branch duct IPMN (BD-IPMN), and mixed type IPMN (MT-IPMN) [6,9], as summarized in Table 1. Each of the IPMN subtypes is characterized by a different risk of progression to malignancy, ranging from 1–38% in BD-IPMN up to 33–85% in MD-IPMN [11]. MD-IPMN is associated with an over 5 mm dilation of the main pancreatic duct (MPD) with no detectable causes for obstruction. In BD-IPMN, over 5 mm dilatation of side ducts with no main duct changes is seen. Finally, MT-IPMN is characterized by the dilation of both the main and side branches of the pancreatic ducts [12]. PDAC is most often located in the pancreatic head (71%) and less frequently in the pancreatic body (13%) and tail (16%) [13]. Similarly, the main localization of IPMN is the head of the pancreas [14].

Histologically, there are four IPMN subtypes, namely, gastric, intestinal, pancreatobiliary, and oncocytic, as summarized in Table 2. The names are derived from the organs whose native tumor features are mimicked by the respective IPMN subtypes [15,16]. Among them, the gastric subtype, usually found in BD-IPMN, presents the lowest lower malignancy risk, while the intestinal and pancreatobiliary subtypes have poorer prognosis and are more often associated with the risk of high-grade dysplasia (HGD) and PDAC [6,9].

Regarding the neoplasia status, IPMN may contain the full spectrum from low-grade dysplasia (LG-IPMN) to high-grade dysplasia (HG-IPMN) and invasive carcinoma (IC-IPMN) [17], as summarized in Table 3. HG-IPMN is defined by pronounced architectural and cytological abnormalities, including atypia and the presence of irregular, branching papillae. In contrast, LG-IPMN displays these features to a much lesser extent [18]. Invasive adenocarcinoma may be the outcome of IPMN progression or occur separately in any part of the pancreas; then, it is called concomitant PDAC [19,20].

The nomenclature of the grade of IPMN malignancy has not been officially established, and many publications use different terms and definitions of malignant lesions. Nevertheless, all grades of IPMN are considered neoplastic, as they represent precursor lesions to PDAC. Grading IPMN is crucial for clinical management, as it directly impacts prognosis and guides treatment decisions—observation vs. surgery. Therefore, despite some terminological inconsistency, this review adopts the following convention: whenever, in relation to IPMN, a benign change is mentioned, it means LG-IPMN, while a malignant change refers to HG-IPMN or IC-IPMN. Similarly, low-risk lesions apply to LG-IPMN, while high-risk changes apply to HG-IPMN or IC-IPMN.

Although the early diagnosis of IPMN and assessment of IPMN-related malignancy, as well as the choice of an adequate management method and timing, are essential, all of them constitute a significant clinical challenge.

## 2. Diagnosis and Management

Most pancreatic cystic lesions (PCLs) are diagnosed incidentally during imaging studies, such as endoscopic ultrasound (EUS), computed tomography (CT), or magnetic resonance imaging (MRI), performed for unrelated indications. Cysts may cause no symptoms or noncharacteristic ones. Those include abdominal distension, acute abdominal pain suggestive of acute pancreatitis, nausea, steatorrhea, weight loss, jaundice, or recent diabetes [21,22,23,24]. MRI, CT, and EUS are generally considered equivalent for diagnosing IPMN, with a slight preference for MRI due to its higher sensitivity in detecting mural nodules—one of the key predictors of malignancy [21,25]. It is important to note that MRI and CT tend to overestimate nodule size, while EUS tends to underestimate it [26].

Despite recent advances, differentiating IPMN from other cystic lesions and accurately assessing malignancy risk through imaging remains challenging (Table 4).

To aid in this process, a set of clinical and radiological predictors of malignancy has been established to guide the risk assessment and management of IPMN. The International Association of Pancreatology Guidelines (IAP), revised in 2024 in Kyoto, highlight the high-risk stigmata (HRS) and worrisome features (WFs) in imaging techniques as the malignancy risk features (Table 5). The HRS are as follows: (1) obstructive jaundice in a patient with a cystic lesion of the head of the pancreas, (2) enhancing mural nodule ≥ 5 mm or solid component, (3) main pancreatic duct ≥ 10 mm, and (4) suspicious or positive results of cytology, if performed [21]. HRS are strong predictive factors of HG-IPMN or IC-IPMN, but do not have satisfactory specificity. The WFs are as follows: (1) acute pancreatitis, (2) increased serum level of CA19–9, (3) new onset or acute exacerbation of diabetes mellitus (DM) within the past year, (4) cyst ≥ 30 mm, (5) enhancing mural nodule < 5 mm, (6) thickened/enhancing cyst walls, (7) MPD ≥ 5 mm and <10 mm, (8) abrupt change in caliber of pancreatic duct with distal pancreatic atrophy, (9) lymphadenopathy, and (10) cystic growth rate ≥ 2.5 mm/year [21].

Specific risk factors differ across various guidelines, as summarized in Table 5. The distinction between HRS and WFs is uniquely specified in the IAP Guidelines; other guidelines do not classify risk factors in this way, although this distinction is widely accepted in clinical practice. Notably, the HRS defined by IAP are consistently recognized across all major guidelines.

Jaundice is recognized as one of the HRS by all major guidelines except AGA. As a symptom of HG/IC-IPMN, it is characterized by a low pooled sensitivity of 26% but a high specificity of 97% [41]. Various studies suggest different thresholds for mural nodule size, though the most commonly adopted cutoff for HRS is ≥5 mm [42]. The diagnostic performance of mural nodules ≥ 5 mm in predicting HG/IC-IPMN shows a sensitivity of 64.6–79.7% and a specificity of 79.8–84.5% [42,43,44,45]. MPD dilatation is included in all guidelines, but with varying cutoff thresholds. The IAP Guidelines classify MPD ≥ 10 mm as an HRS and 5–9 mm as a WF [21], which is followed by European Guidelines [25]. In contrast, the ACR defines MPD 7–9 mm as a WF [46]. In summary, the presence of jaundice, a contrast-enhancing mural nodule or solid component, or MPD dilatation ≥10 mm is associated with PPV for malignancy ranging from 56% to 89% [25].

Other features are considered to have lower predictive value and are categorized as WFs. The interpretation of WFs, however, varies significantly. The strongest consensus exists around the presence of an enhancing mural nodule/solid component, as well as an enlarged cyst size, a dilated MPD, and a cystic growth rate; however, the cutoffs are different, as described in Table 5. Some features, such as a thickened cyst wall, abrupt change in MPD diameter with distal atrophy, lymphadenopathy, abdominal pain, and non-enhancing mural nodules, are mentioned in single guidelines, indicating limited agreement in these areas.

**Table 5:** High-risk factors for malignancy of pancreatic cysts in different guidelines, with their sensitivity and specificity values in predicting malignancy of IPMN [7,21,25,46,47].

Worrisome Factors		Sensitivity	Specificity	Guidelines	References
Jaundice		26–83%	61–97%	IAP	[1,41]
				European	
				ACG	
				ACR	
Enhancing mural nodule or solid component	≥5 mm	64.6–100%	73–87.5%	IAP	[21,42,48]
				European	
				ACG *	
				ACR *	
				AGA *	
	<5 mm	N/A	N/A	IAP	-
				European	
				ACG *	
				ACR *	
				AGA *	
Main pancreatic duct dilation	≥10 mm	28.2–51.7%	78.7–87.5%	IAP	[42,48,49]
				European	
				ACR	
				AGA *	
	≥7 mm	53.80%	80.70%	ACR	[50]
				AGA *	
	>5 mm	54.7–74.8%	58.6–78%	ACG	[49,51]
				AGA *	
	≥5 mm and <10 mm	N/A	N/A	IAP	-
				European	
				AGA *	
Positive cytology		28.7–64.8%	84–94%	IAP	[37,38,39,40]
				European	
				ACG	
				AGA	
Acute pancreatitis		32–42.6%	86–86.1%	IAP	[41,48]
				European	
				ACG	
New-onset or worsening diabetes		46%	83%	IAP	[41]
				European	
				ACG	
Increased serum level of CA 19-9	>37 U/mL	41–74%	85–96%	IAP	[21,41,48]
				European	
				ACG	
Cyst diameter	≥40 mm	N/A	N/A	European	-
	≥30 mm	56.1–64%	53.7–69%	IAP	[41,51]
				ACG	
				ACR	
				AGA	
Thickened/enhancing cyst walls		23–38.5%	89.7–95%	IAP	[41,51]
				ACR	
Abrupt change in caliber of pancreatic duct with distal pancreatic atrophy (IAP)		19.30%	95.90%	IAP	[51]
focal dilation of pancreatic duct concerning for MD-IPMN or an obstructing lesion (ACG)				ACG	
Lymphadenopathy		5.2–20%	93–99.6%	IAP	[41,51]
Cystic growth rate	≥5 mm/year	56%	97%	European	[52]
	>3 mm/year	N/A	N/A	ACG	-
	≥2.5 mm/year	60.90%	70.30%	IAP	[51]
Abdominal pain		N/A	N/A	European	-
Non-enhancing mural nodule		N/A	N/A	ACR	-

* They do not specify a cutoff point for size. Abbreviations: IAP, International Association of Pancreatology; ACG, American College of Gastroenterology; ACR, American College of Radiology; AGA, American Gastroenterological Association.

Early detection of IPMN is particularly important. In the case of detecting a malignant lesion, resection is advised. It has been shown that the survival of patients operated on for IC-IPMN is several times higher than that of those operated on for PDAC (median 76.6 vs. 26.54 months; 5-year overall survival rate: 65.4% vs. 14.2%, respectively) [53]. On the other hand, in cases where benign IPMN is diagnosed, the patient can be placed under surveillance over time, and surgery can be implemented if malignant features appear, which significantly reduces mortality. This also leads to a decrease in the number of unnecessary surgeries.

**Figure 1:**
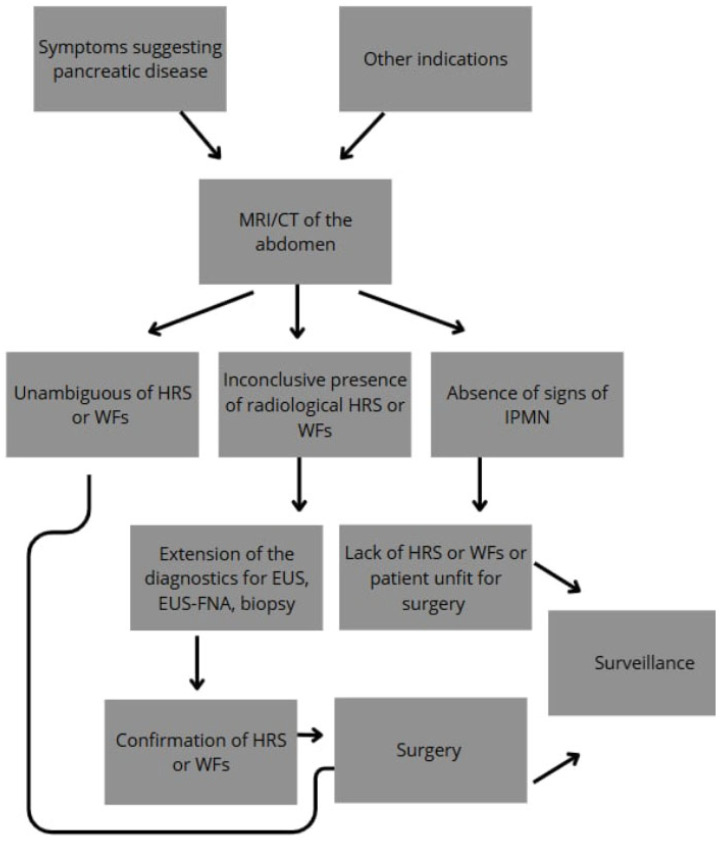
Diagram summarizing the diagnostic and management algorithm for IPMN.

The high-risk features described above constitute the basis for further management of the patient (Figure 1). In general, patients with a lesion most likely to be malignant (HGD or IC) should undergo pancreatic resection. The others are considered for CT, MRI, or EUS surveillance [9]. According to the IAP Guidelines, surgery should be proposed when any of the HRS are present or when additional conditions are met, such as multiple WFs, repeated acute pancreatitis likely to aggravate the patient’s life quality, and if the patient is fit for surgery. It has been shown that the more WFs that are present, the higher the risk of malignancy in IPMN. The presence of one WF increases the malignancy risk by 22%, two WFs by 34%, three by 59%, and four or more by 100% [54]. Decisions about surgery should also consider the patient’s general condition, comorbidities, preferences, and life expectancy. An optimal surgical strategy for IPMN includes radical pancreatectomy with lymphadenectomy or organ-preserving pancreatectomy, depending on whether the lesion is suspected to be invasive or not [21].

A major challenge is accurately selecting BD-IPMN cases for surgery. This subtype accounts for about 80% of IPMNs and carries the lowest risk of progression to cancer (1–48%) [11,21]. However, this risk may be overestimated because many benign BD-IPMNs were managed conservatively through observation rather than surgery [21].

In the absence of any HRS or WFs, the patient should be placed under surveillance. The frequency of follow-up examinations (preferred MRI, CT, and/or EUS) depends on the size of the largest initial lesion. For cysts smaller than 20 mm, the frequency is once after 6 months, then every 18 months. For cysts measuring 20–30 mm, the frequency is twice at 6-month intervals and then annually. For cysts larger than 30 mm, the frequency is every 6 months [21]. If there is no progression, surveillance can be stopped after 5 years. However, if the size of the cyst increases, the HRS and WF assessment should be repeated, and surgery should be reconsidered [21].

Surveillance is also recommended after surgery: if the excised lesion is invasive, the same surveillance as in PDAC is necessary. If the excised lesion is not invasive, regular imaging tests are performed as part of the surveillance: every 6 months, if HGD is present or there is a family history of pancreatic cancer; otherwise, every 12 months. If the surveillance proceeds correctly, which means no change over time for a small cyst (<2 cm) without HRS and WFs, it can be discontinued after 5 years. Also, surveillance should be stopped when a patient is unfit for surgery or has a life expectancy of <10 years [21]. The optimal methods for surveillance are MRI, physical examination, and monitoring of tumor markers: carcinoembryonic antigen (CEA), CA 19-9, and diabetes—hemoglobin A1c. CT and EUS should be considered as a secondary option for surveillance when changes are observed in the MRI [21]. European Guidelines state that MRI is the preferred method for surveillance; however, CT and MRI have similar accuracy for cyst assessment [25]. Also, the AGA Guidelines place MRI first, highlighting the drawbacks of other methods: radiation in CT and the relatively high invasiveness of EUS [47]. Indications for surgery and surveillance differ between major guidelines, which are summarized in Table 6.

## 3. MRI Imaging in Detecting High-Risk Malignancy in IPMN

Some studies showed that new MRI techniques and magnetic resonance cholangiopancreatography (MRCP) can improve the detection of contrast-enhanced mural nodules. The value of 1.5T MRI with diffusion-weighted imaging (DWI) was evaluated in patients who underwent pancreatic surgery for IPMN. MRI was performed prior to surgery and showed a correlation between the presence of contrast-enhanced mural nodules ≥5 mm and pathologically confirmed high-grade dysplasia, with a sensitivity of 90.77% and a specificity of 84.62% [27]. Another retrospective study was performed with 3T MRCP. Imaging was completed before surgical treatment in 73 patients with pathologically proven IPMN. MRCP showed a sensitivity of 94.1% and a specificity of 87.2% for detecting enhancing mural nodules ≥5 mm in patients with malignant IPMN [32].

On the other hand, MRI has poorer results in detecting MPD dilation. In one study, 3T MRCP was performed in 151 patients prior to IPMN resection. Among the patients studied, 68.9% had low-grade dysplasia (LGD) and 31.1% had HGD or invasive carcinoma. In the study, 51.7% of the patients were classified preoperatively as MD/MT-IPMN and 48.3% as BD-IPMN. The cutoff value of the main pancreatic duct diameter considered malignant in the mentioned study was 1.0 cm. A comparison of the results to the histopathological findings showed a sensitivity of 38.3% and a specificity of 93.1% for MPD dilation in all types of IPMN. The relatively low efficacy of MRCP was due to the BD-IPMN results. Particularly, there was no difference in the MPD diameter between low-risk and high-risk disease. Univariate analysis of MRI results, like the MPD, mural nodules, cyst size, and localization, showed that mural nodules were the only significant predictor for high-risk IPMN in the study. The best results were obtained with an MPD diameter of 0.77 cm, with a sensitivity of 61.7% and a specificity of 87.5% [33].

MRI could also include detecting proton density fat fraction (PDFF). PDFF is an imaging biomarker that allows the quantitative assessment of adipose tissue, based on MR signal intensity [55]. In one study, pancreatic fat induced chronic inflammation leading to the development of pancreatic cancer, suggesting that pancreatic fatty infiltration is a risk factor for PDAC [56]. Twenty-four patients with PDAC underwent 3T MRI prior to pancreatectomy. The histologic pancreatic fat fraction was defined as the percentage of intraparenchymal fat of the total pancreatic parenchyma. PDFF was measured by image analysis software. MRI-PDFF was measured manually by a radiologist from the fat fraction images. Images were obtained from the region of interest (ROI) and the estimated resection line. Histological fat friction was correlated with MRI-PDFF (*p* < 0.01). The median MRI-PDFF in PDAC was 9.09%, and in the control group, it was 3.42%. PDFF was significantly higher in the PDAC group (*p* < 0.01), showing that higher PDFF is correlated with PDAC [56]. A 3T MRI study was performed in IC-IPMN, IPMN, and subjects with no pancreatic lesions (controls). It was demonstrated that PDFF is significantly higher in IC-IPMN than in IPMN and controls (*p* < 0.001). There was no PDFF difference between the IPMN group and the normal pancreas group (*p* = 0.916) [57]. PDFF in the pancreas was also elevated in obesity with BMI >85%, in pancreatic exocrine insufficiency or chronic pancreatitis, and in pancreatic endocrine insufficiency [58].

In another new technique, radiomics, a number of critical quantitative features are extracted from digital images. The detailed, high-dimensional databases created in this way serve as a starting point for mathematical analysis, enabling the prediction of a certain clinical output, e.g., the risk of malignancy in IPMN, based on the difference in radiological pictures between healthy patients and a group with pathologically proven IPMN [34,59]. In a multicenter study of 202 patients, the radiomic MRI model with pathologically diagnosed BD-IPMN was evaluated. For better validation, patients were divided into training cohort groups (103 patients) and two external validation cohorts (first validation with 48 patients and second validation group with 51 patients). The radiomic model showed good results in predicting IPMN histological grade (LGD, HGD, and IC). The AUC in the training group was 0.836; in the first validation group, the AUC was 0.811, and in the second validation group, the AUC was 0.822. The combined nomogram of radiomic and MPD diameter, together with the CA 19-9 level, showed even better results—an AUC of 0.903, a sensitivity of 73.4%, and a specificity of 94.8% for the training group; an AUC of 0.884, a sensitivity of 90.0%, and a specificity of 79.0% for the first validation group; and an AUC of 0.876, a sensitivity of 85.7%, and a specificity of 83.7% for the second validation group. These data confirm that combined nomograms may be a very valuable diagnostic tool for predicting the histological grade of IPMN [28]. In another retrospective study, 60 patients with pathologically proven IPMN underwent CT and 1.5T MRI with MRCP prior to surgical intervention. The MRI pictures were analyzed by two radiomics systems. The diagnostic performance of radiomics in predicting the malignant potential of IPMN was based on the international consensus Fukuoka guidelines for the management of IPMN from 2017. The MRI radiomics with LR (logistic regression) showed a sensitivity of 91.3%, a specificity of 78.4%, and an AUC of 0.879 for predicting malignant IPMN. The MRI radiomics with SVM (support vector machine) showed a sensitivity of 95.7%, a specificity of 81.1%, and an AUC of 0.940 [60].

## 4. Computed Tomography in Detecting Malignancy in IPMN

Contrast-enhanced CT and MRI present similar diagnostic performance for the differentiation of malignant and benign IPMNs, according to the 2017 International Consensus Fukuoka Guidelines [29,30]. In a retrospective study, 175 patients underwent preoperative CT and MRI and were assessed for the presence of HRS. CT predicted malignant IPMN with a sensitivity of 79.5% and a specificity of 67.8%, while MRI had a sensitivity of 86.4% and a specificity of 64.4% [29]. In a similar study of 86 patients, which defined malignant IPMN as the presence of four or more WFs or at least one HRS, CT predicted malignancy with a sensitivity of 86% and a specificity of 74%, while MRI had a sensitivity of 89% and a specificity of 83%, which was not significantly different [30].

Several studies confirmed the high diagnostic performance of preoperative CT-based radiomics analysis in stratifying the risk of malignant IPMN. In a large study of 408 patients by Tobaly et al. [34], a multivariate model of radiomics-only data based on CT features, previously determined in a univariate analysis as significant, differentiated malignant from benign tumors in two cohorts with an AUC of 0.71–0.84, a sensitivity of 69–82%, and a specificity of 57–74%. These radiomic features included differences in shape, matrix gray saturation, neighboring gray tone difference matrix, and others. Adding variables representing indications for surgery in the form of HRS and WFs, according to the 2017 ICG Fukuoka Recommendations and 2018 European Recommendations, gave comparable values: an AUC of 0.75–0.83, a sensitivity of 69–80%, and a specificity of 65–72%. In addition, Chakraborty et al. [35] combined various radiologic features (for example, high- or low-intensity pixels representing, respectively, solid components in cysts or hypoattenuated dilations in parenchyma, and image texture features based on spatial pattern and contrast) with five clinical variables (age at resection, cyst size, presence of a solid component, presence of symptoms, gender). As a result, a high AUC of 0.81, a sensitivity of 84%, and a specificity of 70% were obtained. Since BD-IPMN is often benign, the correct assessment of BD-IPMN malignancy before potential resection is particularly important [34]. In a group of patients with postsurgical diagnosis of BD-IPMN, the model based only on specific quantitative imaging features—extracted from CT images and designed to provide an in-depth analysis of mural nodules—turned out to be more accurate than the clinical model alone (age, cyst size, presence of a solid component, symptoms, gender) (AUC 0.76 vs. 0.67). The combination of both imaging and clinical features achieved slightly better performance: an AUC of 0.79 with a maximal sensitivity of 71% and a specificity of 82% [61]. Lou et al. [36] extracted radiomics features (first-, second-, and higher-order) independently in the arterial and venous phases. Interestingly, the venous phase radiomics features appeared to be more accurate than the arterial phase in the prediction of the malignancy of IPMN. An integrated model of clinical and venous phase radiological features achieved high predictive performance: a sensitivity of 90%, a specificity of 74%, and an AUC of 0.904. The authors noted that, although the arterial phase model included unique high-dimensional radiomics features that were individually less effective than those from the venous phase, these features may still contribute value in a multiphase radiomics assessment [36].

Kim et al. [62] demonstrated that CT may be useful in predicting high-grade pancreatic intraepithelial neoplasia (HG-PanIN) in patients with IPMN. The study included 251 patients who underwent partial pancreatectomy for IPMN and had a pathologically confirmed PanIN grade. Although PanIN is not visible in imaging, it has been shown that common CT features, such as mural nodule size (with a cutoff value of ≥ 15 mm) and abrupt MPD change with distal pancreatic atrophy, were significant predictors of HG-PanIN in multivariate analysis. Furthermore, HG-PanIN was significantly associated with tumor recurrence in pancreatic remnants. In addition, an abrupt MPD caliber change and lymphadenopathy were found to be highly predictive for HGD or IC-IPMN [63].

Notably, CT imaging can be valuable in preoperative differentiation of already existing IPMN-based cancers, including colloid carcinoma and tubular adenocarcinoma. This differentiation is particularly important due to the different prognosis, which is better for colloid carcinoma. The authors of one study identified CT findings that correlated with this cancer type, confirmed at surgery. The most specific were calcification of cystic lesions (specificity 97.9%) and fistulas (specificity 100%). In addition, the features strongly associated with colloid carcinoma were cyst location in the pancreatic head, uncinate process, or neck of the pancreas, septation of cystic lesions, the largest cystic lesion diameter of ≥ 28 mm, and mural nodules in cystic lesions or the MPD. In contrast, tubular adenocarcinoma was more strongly associated with extracystic or extracellular solid mass and an abrupt change in MPD caliber due to an adjacent extracystic or extraductal solid mass pressure. This latter feature was the most effective in differentiating between those two cancer types, with a sensitivity of 81.3% and a specificity of 92.3% [64].

**Figure 2:**
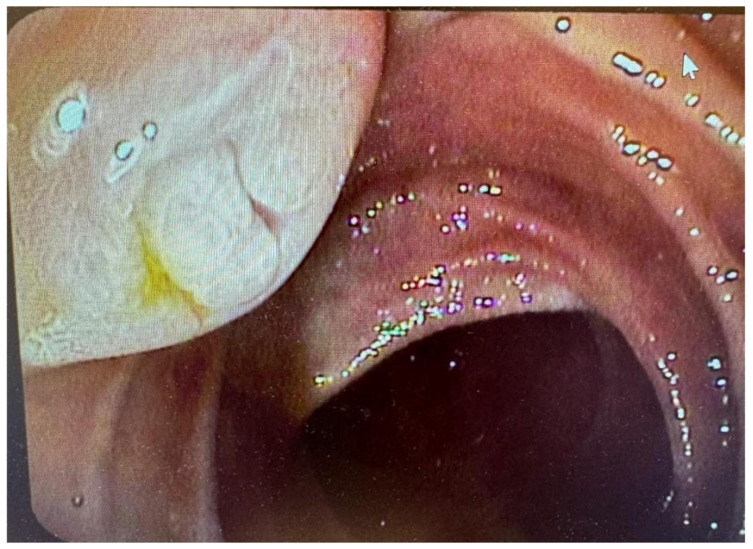
Endoscopic view of the major duodenal papilla with the fish mouth sign. Source—B. Włodarczyk, Dept of Digestive Tract Diseases, Medical University of Lodz, Poland.

Finally, the fish mouth ampulla sign is a well-known IPMN sign in duodenoscopy (Figure 2), but it was recently visualized on CT as an uninterrupted column of water attenuation material running from the MPD to the duodenal lumen. This sign on CT demonstrates extremely high specificity (up to 100%) in the diagnosis of MD-IPMN or MT-IPMN. Nevertheless, its presence is not correlated with malignancy risk [65].

The efficacy of CT in detecting malignancy in IPMN based on the abovementioned studies is summarized in Table 7.

MRI and CT should be viewed as complementary, as they capture HRS features in different ways. PDFF, a marker available through MRI, has recently emerged as an indicator of inflammation and pancreatic steatosis, both of which are risk factors for cancer development in IPMN. Nevertheless, the most significant potential for advancement lies in the integration of information technology, mathematics, and artificial intelligence (AI). Radiomics enables the extraction of quantitative features from MRI and CT images that are not discernible through conventional radiological analysis yet may hold substantial predictive value in statistical models. It may also help to avoid human error due to a lack of time or different expertise. Combining radiomic image features with clinical data into a unified model has proven even more effective. This integrative approach may serve as a foundation for developing robust predictive tools, such as nomograms, to improve clinical risk stratification in patients with IPMN.

## 5. Endoscopic Ultrasound in Detecting Malignancy in IPMN

**Figure 3:**
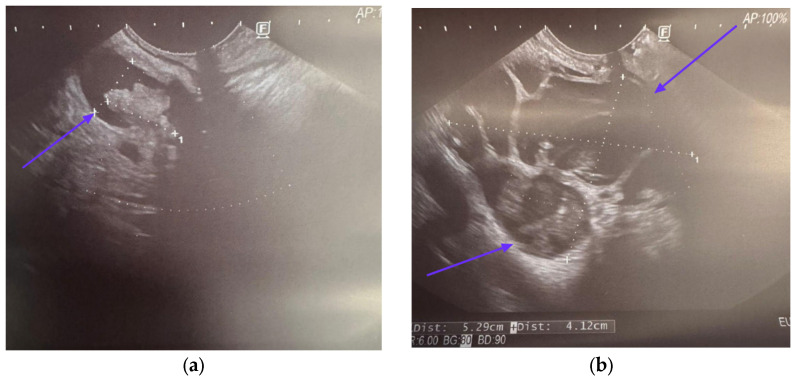
IPMN in EUS. (**a**) MPD dilated to 14 mm; (**b**) pancreatic tumor. Source—B. Włodarczyk, Dept of Digestive Tract Diseases, Medical University of Lodz, Poland.

The European Guidelines recommend EUS as an adjunct to other imaging methods when the clinical or radiological suspicion is not cleared of malignancy [25]. A similar recommendation appears in the latest IAP Kyoto Guidelines from 2024, which introduced an important change in the approach to EUS. In particular, EUS, including endoscopic ultrasound fine-needle aspiration (EUS-FNA) and contrast-enhanced harmonic (CH-EUS), was endorsed for evaluating HRS and WFs due to its ability to confirm the presence and size of mural nodules and to enable cyst fluid sampling or biopsy of solid components (Figure 3) [21]. In the latest literature, EUS is equivalent to MRI and CT in detecting HGD and IC in IPMN [66].

Several drawbacks of EUS are acknowledged. They include high dependence on the experience of the operators, differences in the amount and quality of fluid collected during EUS-FNA, and lower replicability of the results. A 2021 meta-analysis of 28 studies involving 1812 patients found that EUS represented the poorest accuracy in distinguishing benign from malignant IPMN among all studied imaging techniques: CT, DWI, EUS, MRI/MRCP, and PET/CT. The results showed a pooled sensitivity of 60%, a pooled specificity of 80%, and an AUC of 0.79. This meta-analysis took into account studies in which malignancy was confirmed with a postsurgical specimen, biopsy pathology, or cytology [31]. However, there has been significant progress in EUS technology recently, with new EUS-assisted techniques, like CH-EUS and EUS-guided needle-based laser confocal endomicroscopy (EUS-nCLE). Recent studies have shown that the sensitivity of CH-EUS in the diagnosis of mural nodules is above 90%, which is higher than conventional EUS and CT [67,68]. Conventional EUS and CH-EUS present valuable advantages in the assessment of mural nodules in IPMN, including high spatial resolution imaging and better differentiation between intracystic mural nodules and mucous clots, due to mural lesion vascularity visualization [42,68]. Moreover, CH-EUS is able to evaluate MPD involvement in BD-IPMN as well as the evidence of mural nodules or solid components infiltrating the MPD. MPD involvement is defined as the presence of papillary projections extending into the MPD. In a study of 166 patients who underwent resection for BD-IPMN, CH-EUS achieved a sensitivity of 83.5% and a specificity of 87% in detecting MPD involvement [69].

### 5.1. EUS-Guided Fine-Needle Aspiration

EUS-FNA improves diagnostic accuracy in distinguishing between mucinous and non-mucinous PCN, as well as between benign and malignant lesions [25]. However, EUS-FNA is an invasive procedure, albeit relatively safe. Therefore, it should be reserved for cases where imaging results are inconclusive, further diagnostic clarification is necessary, and its result (e.g., neoplasia in cytology) is expected to influence the patient’s management. In cases where imaging strongly suggests malignancy, such as the presence of HRS, surgical intervention is typically undertaken without prior EUS-FNA [21,25].

EUS-FNA is considered as a relatively safe procedure, with a 3.4% risk of complications [25]. In a meta-analysis of 5124 patients with PCL comprising the EUS-FNA-associated adverse effects, morbidity and mortality were 2.66% and 0.19%, respectively. The complications included mainly pancreatitis, pain, and infection [70]. There was also a small risk of peritoneal cancer cells dissemination due to EUS-FNA [21]. The approximate cost of the EUS-FNA procedure, based on Khoury and Sbeit, was USD 1029 [71].

#### 5.1.1. Cyst Fluid Analysis

EUS-FNA enables the acquisition of cyst fluid for cytology, the presence of mucin, CEA, amylase, glucose, and KRAS/GNAS mutations. A cyst fluid CEA (cutoff value > 192 ng/mL) has a sensitivity of 56–58% and a specificity of 87–96% for identifying mucinous cysts [72,73], while glucose (cutoff value < 50 mg/dL) has values of 91% and 86%, respectively [73]. Amylase presence in cyst fluid shows communication with the MPD [74]. However, amylase levels are not specific to any particular type of pancreatic cyst. For instance, it can vary widely in IPMN, ranging from 1119 to 38,290 IU/L (median 5090 IU/L) [75]. Amylase is useful primarily for excluding a pseudocyst, with a high specificity of 98% when the level is below 250 IU/L [76].

The most common genetic mutations detected in IPMN are KRAS (found in 40–70% of cases) and GNAS (40–65%) [77,78]. GNAS mutations are mainly found in intestinal types of IPMN and in 50% of gastric types [78]. A meta-analysis of KRAS mutations in pancreatic cystic fluid for diagnosing HGD or IPMN-driven adenocarcinoma showed a sensitivity of 54%, a specificity of 51%, and an AUC of 0.51. A meta-analysis of GNAS mutations showed a sensitivity of 29%, a specificity of 46%, and an AUC of 0.29 in detecting HGD or IPMN-IC [40].

#### 5.1.2. Cytology

Cytology obtained via EUS-FNA is characterized by a low diagnostic sensitivity of 28.7% (ranging from 4.8% to 61.5%) in detecting malignant PCL [37], but a positive result has high specificity and a significant impact on management [21]. In a study by Wesali et al. [79], 58 patients underwent surgery for suspected PCL. The combination of EUS, cytology, and CEA levels (cutoff value >1000 ng/mL) was able to detect PCL with HGD or IC, yielding a sensitivity of 33% and a specificity of about 75%.

To improve cytology sensitivity, the Papanicolaou Society of Cytopathology developed standardized guidelines. They included unified indications for EUS-FNA, detailed pathology descriptions, images, ancillary testing, and post-biopsy treatment and management, alongside a six-level terminology classification: (1) nondiagnostic, (2) negative, (3) atypical, (4) neoplastic: benign and other, (5) suspicious, and (6) positive/malignant [80,81]. This multidisciplinary approach resulted in a reduction in the number of atypical and nondiagnostic results, as well as enabled more definitive diagnoses of neoplasia [81,82,83,84]. The use of these guidelines has presented varied diagnostic sensitivity, specificity, PPV, and NPV of EUS-FNA in detecting malignant lesions, with values of 48.3–100%, 66.7–100%, 88–100%, and 33.3–100%, respectively [83,85,86,87,88]. In a study of 41 patients after resection due to IPMN, the preceding cytology results strongly correlated with postsurgical pathology (sensitivity 67%, specificity 94%) [89]. The WHO Reporting System for Pancreatobiliary Cytopathology recently revised these guidelines, introducing a seven-tiered system with categories based on cytological features and ancillary tests. Each of them represents a different malignancy risk: (1) insufficient/nondiagnostic, 5–25%, (2) benign, 0–15%, (3) atypical, 30–40%, (4) pancreatic neoplasm of low risk/low grade (PaN-low), 5–20%, (5) PaN of high risk/high grade (PaN-high), 60–95%, (6) suspicious for malignancy, 80–100%, and (7) positive for malignancy, 99–100% [84]. The WHO system retained the previous multidisciplinary approach but changed the classification of neoplasia. The “neoplastic” category is now divided into PaN-low and PaN-high, with the majority comprising IPMNs and other mucinous cystic neoplasms. This enables a more accurate assessment of the malignancy risk of LG-IPMN and HG-IPMN [84]. These revised guidelines standardize cytological interpretation, risk assessment, additional biochemical and molecular tests, and further management, aiming to improve communication between clinicians and cytopathologists.

According to the 2024 Kyoto Guidelines, suspicious or positive results of cytology are classified as HRS and should be considered a reason to pursue surgery [21].

### 5.2. EUS-Guided Through-the-Needle Biopsy

Endoscopic ultrasound through-the-needle biopsy (EUS-TTNB), also known as EUS–microforceps biopsy, is a diagnostic tool that provides histological specimens from the cystic lesion, such as the cyst wall, septa, or mural nodules [90]. Biopsy forceps are passed through the EUS-FNA needle under EUS guidance, and histological samples are collected from the different parts of the cyst or suspected intracystic masses. EUS-FNA is disturbed due to the low cellularity of the samples. The CEA level available from EUS-FNA does not correlate with the level of dysplasia. EUS-TTNB allows the evaluation of cyst type and dysplasia grade. EUS-TNNB gives macroscopically visible, abundant samples [91,92]. A meta-analysis of 3641 patients with PCLs showed that the EUS-TTNB method has sensitivity and specificity values in differentiating mucinous from non-mucinous lesions of 87% and 83%, respectively. For malignant cysts diagnosis, the sensitivity is 90% and the specificity is 95% [42,93].

A single-center study of EUS-TTNB and EUS-FNA was performed in 57 patients. Different stages of IPMN were detected in EUS-TTNB. Cytological material from EUS-FNA showed IPMN cells only in 18 of 57 patients [92]. In another multicenter study of 111 patients with PCL, EUS-FNA and EUS-TTNB were performed. Mucinous cysts were diagnosed with EUS-TTBN in 61 of the cases, and only 11 cases were detected with EUS-FNA (*p* < 0.001). Surgical pathology was consistent with EUS-TTNB in all IPMN cases operated at 100%. In terms of accuracy, EUS-FNA showed IPMN only in one of nine cases. EUS-FNA did not show any dysplasia grade in later-detected HGD or IC-IPMN. EUS-TTNB dysplasia grade was correlated with postsurgical results in four of five cases [94]. The differentiation of mucinous and non-mucinous lesions in EUS-TTNB was performed on a large group of patients. In contrast, determining the malignant status of IPMN was conducted on a very limited number of patients. Although the results are promising, it is necessary to confirm and validate the clinical output of EUS-TTNB in larger-scale research.

The advantages of EUS-TTNB are the possibility of showing specific cyst types and the grade of dysplasia. Endoscopic ultrasound–fine-needle biopsy AGA 2015 (EUS-FNB) is a diagnostic tool for increasing tissue accusation by developing needles. EUS-FNB is believed to obtain tissue samples with better preserved tissue architecture, allowing for pathological evaluation, more accurate molecular diagnostics, and immunohistochemical stains [95,96,97]. However, EUS-FNB produced adequate specimens for histological evaluation of PCLs only in 46.1% [98]. EUS-FNB should be performed in lesions suspected of IPMN with the presence of mural nodules, thick septation walls, or mucin balls within the cyst, especially if other methods are not available [97]. Among EUS-FNB techniques, modified wet-suction provides better tissue preservation, although it carries a higher risk of blood contamination compared to the slow-pull technique. Nevertheless, both techniques have shown comparable diagnostic accuracy in evaluating solid lesions ≥ 1 cm, whether of pancreatic origin or not [96,99]. If HGD or IC-IPMN is detected in EUS-TTNB, surgical treatment should be performed [21]. The disadvantage of EUS-TTNB is the special training and necessary experience required. Some adverse effects may occur within a range of 2–23% and seem higher than EUS-FNA. The most common are intracystic bleeding, mostly without clinical implications. The pooled rate for acute pancreatitis was around 3%. Mostly, acute pancreatitis had a mild clinical course, but some severe ones are possible. Infections are rarely seen, and antibiotic prophylaxis does not increase the infection rate. The severe adverse events pool rate is around 1.1% mostly due to severe acute pancreatitis [100]. The cost of EUS-TTNB is pretty high due to the association with EUS-FNA [101].

### 5.3. EUS-Guided Needle-Based Confocal Laser Endomicroscopy

Endoscopic ultrasound-guided needle-based confocal laser endomicroscopy (EUS-nCLE) is a novel technique that shows real-time pancreatic cyst epithelial lining patterns in a way close to microscopic. It can assess changes in vesicular architecture, connective tissue, and cellular components in the mucous layer. It is performed under classic EUS but requires intravenous photosensitizing contrast prior to imaging. A laser probe is introduced into the target cyst through the needle under the control of EUS. The laser probe touches the cystic wall, and it is gently moved around to different areas of the internal lining, visualizing in vivo patterns corresponding to particular histopathology images. Different types of pancreatic cysts show various endomicroscopy imaging patterns, which may indicate a specific type of cyst and even the grade of dysplasia in IPMN [102,103,104]. In endomicroscopy, IPMN shows finger-like papillary projections. The grade of dysplasia can be determined by the epithelium thickness. LGD in EUS-nCLE is characterized by a thin layer of epithelium. HGD has a significantly thicker lining, and it is much darker, which seems to suggest many layers and nuclear abnormalities [102]. Some studies were performed to determine the sensitivity and specificity of EUS-nCLE. In 26 patients, it showed sensitivity, specificity, and accuracy values for the detection of HGD or IC in IPMN of 90%, 73%, and 83%, respectively [105]. In addition, with this method, HGD or IC was detected with 87% sensitivity, 100% specificity, and an AUC of 0.95 [105]. The final diagnosis was based on resected specimen pathology in 24 patients and on metastatic liver lesion biopsy in 2 patients [105]. In another study, 60 patients with BD-IPMN underwent EUS-nCLE. The EUS-nCLE results were analyzed by experienced endosonographers and by functional EUS-nCLE-based AI algorithms. The EUS-nCLE videos were analyzed only by the video from the nCLE, with additional data from the revised Fukuoka HRS and WFs. The results were compared to the surgical pathology. The detection of HGD or IC in BD-IPMN by human observers without revised Fukuoka data had a sensitivity of 58.2%, a specificity of 58.8%, an accuracy of 58.5%, and an AUC of 0.59. Adding the revised Fukuoka HRS or WFs improved the specificity to 72%, while the sensitivity and accuracy were similar to the data without that information, with a sensitivity of 56.7%, an accuracy of 62.6%, and an AUC of 0.64. The AI algorithm showed a sensitivity of 87%, a specificity of 54.1%, an accuracy of 66.7%, and an AUC of 0.7 for determining HGD or IC without the revised Fukuoka HRS of WFs. Adding the revised Fukuoka HRS and WFs to the algorithm showed a specificity of 78.3%, a sensitivity of 78.4%, an accuracy of 78.3%, and an AUC of 0.85 [106]. Another study used a multicenter database with the 76 most common pancreatic cyst EUS-nCLE images, among them, 37 IPMNs. It showed sensitivity, specificity, and accuracy values for mucinous vs. non-mucinous cyst differentiation of 95.2%, 94.2%, and 94.8%, respectively. The sensitivity, specificity, and accuracy of differentiating IPMN from other PCLs were 84.4%, 88.0%, and 86.2% [107]. All studies were performed on a small number of patients. Further studies should involve a larger group of patients from various centers. EUS-nCLE is promising in the determination of cyst type, but currently, it is not widely available; moreover, it requires special endoscopic training. Experienced operators have high diagnostic accuracy in determining mucus from non-mucinous lesions, specific cyst types, and even the grade of dysplasia. EUS-nCLE may be a useful adjunct in visually targeted biopsy. Some studies already show that combining EUS-nCLE and EUS-TTNB in PCLs is safe [108]. EUS-nCLE equipment is expensive and requires special maintenance. The initial cost of the EUS-nCLE system is approximately USD 100,000–150,000. It also requires miniprobe changes. Monirobes are reusable approximately 10 times and cost around USD 4000. EUS-nCLE procedures may cause complications, such as acute pancreatitis (2.28%), intracystic bleeding, abdominal pain, and infection [102,109,110].

The efficacy of EUS-nCLE diagnosis is summarized in Table 8.

## 6. New Emerging Markers for Malignancy in IPMN

This section describes potential new biomarkers obtained from cyst fluid or tissue specimens during surgery or an EUS procedure, which may be useful in the future. Importantly, the techniques described in this subsection have not yet found application in clinical practice. Although they have shown good results in diagnosing malignancy in IPMN in single studies on small patient groups, further research is needed to confirm these findings. It should also be emphasized that the techniques presented here, due to their high technological advancement, may be useful especially in large, specialized centers, but less so in smaller facilities.

Mucins (MUCs) are highly glycosylated proteins released into a cyst from the epithelium [74], and the positive correlation between MUC protein expression and the degree of dysplasia has been shown [111]. Stiles et al. [112] found that the membranous expression of MUC13 in IPMN tissue was significantly higher in malignant than in benign IPMN, which was detected in the immunohistochemistry of surgical specimens. In a logistic regression model, the mRNA expression of MUC4 in cyst fluid, assessed using quantitative polymerase chain reaction (PCR), showed a strong association with malignant IPMN in samples with a confirmed postsurgical diagnosis of IPMN from institutional databases and repositories [113].

A panel of nine miRNAs (miR-18a, miR-24, miR-30a-3p, miR-92a, miR-99b, miR-106b, miR-142-3p, miR-342-3p, miR-532-3p) present in cyst fluid, proposed by Matthaei et al. [114], showed high efficacy in assessing the malignancy of pancreatic lesions with a sensitivity of 89%, a specificity of 100%, and an AUC of 1. Analyzing the same panel of nine miRNAs, Utomo et al. [115] confirmed its high specificity in estimating the risk of malignancy (HG-IPMN, IC-IPMN, mucinous cystic neoplasm). In a cohort with histological confirmation, a logistic regression model calculated the risk of malignancy, which, at an optimal cutoff point of 25%, had a sensitivity of 66.7% and a specificity of 89.7%. Shirakami et al. [116] evaluated another panel of six miRNAs (miR-711, miR-3679-5p, miR-6126, miR-6780b-5p, miR-6798-5p, miR-6879-5p) using RT-PCR, whose levels were significantly elevated in cyst fluid obtained from intraductal papillary mucinous carcinoma compared to intraductal papillary mucinous adenoma. The final diagnoses were obtained with the histopathology of the postsurgical specimen. Importantly, miRNA molecules may prove unstable in contact with pancreatic juice or bile, which can be a major drawback in routine miRNA evaluation.

Another line of research evaluates the metabolomic and lipidomic profiles for detecting malignancy. Metabolic reprogramming is a key characteristic of cancer. In simplified terms, it involves changing metabolic processes to increase the production of macromolecules, such as lipids, proteins, and nucleic acids, which are supposed to support cell growth and proliferation [117]. In particular, the development and advancement of pancreatic cancer are linked to the modifications in blood metabolic profiles assessed in targeted mass spectrometry [118,119,120]. Gaiser et al. [121] performed an analysis of 100 metabolites and over 1000 lipids in cyst fluid and blood of patients undergoing resection. Patients with IPMN had significant lipid pathway alterations compared to those with serous cysts. Integrated metabolome and lipidome data were extremely effective in distinguishing between mucous and serous cysts (balanced accuracy 100%, sensitivity 100%, specificity 100%) and slightly less effective in diagnosing LG-IPMN or HG-IPMN/IC-IPMN (balanced accuracy 89–91%, sensitivity 88–89%, specificity 91–92%). In cyst fluid, choline, spermidine, phosphoethanolamine, betaine, 2-aminoisobutyrate, and 4-L-hydroxyproline demonstrated the most statistically significant differences between mucinous and serous cyst groups. The most discriminative compounds in plasma were glycine, serine, 2-aminoisobutyrate, dimethylglycine, and glyceraldehyde. Although these analytes were identified based on their strong discriminatory power (the highest variable importance in projection scores), the authors did not report specific cutoff values for their concentrations [121]. In most of those studies, the final diagnosis was based on postsurgical pathologic evaluation.

Finally, the murine monoclonal antibody Das-1, developed to interact with colon epithelial protein (CEP) expressed in normal human epithelial cells of the colon, was shown to interact with antigens present in HG-IPMN and other mucinous lesions. Reactivity with Das-1 was assessed by the sandwich ELISA assay. The ELISA plate was coated with Das-1 antibodies and cyst fluid samples, normalized by protein amount, or enriched CEP protein for the positive control [122]. Positive reactivity of Das-1, with a cutoff value of optical density (OD) ≥ 0.104, successfully diagnosed mucinous lesions with a sensitivity of 88.2% and a specificity of 98.5%. Also, Das-1 presented a notable value in predicting high-risk IPMN, with a sensitivity of 88.3% and a specificity of 92.7% [123].

## 7. Pancreatoscopy

Pancreatoscopy (POP) is a diagnostic tool that allows the direct visualization of the MPD. At duodenoscopy, through the working channel of the apparatus, a smaller scope is passed into the MPD via the major papilla. The smaller endoscope allows probe movement, has a washing canal, and allows for therapeutic lithotripsy. Another system used for POP includes only one ultra-slim endoscope with an inflatable balloon, which dilates and stabilizes the MPD and allows an ultrathin gastroscope to enter the MPD [124]. In both cases, a targeted biopsy may also be performed. Pancreatoscopy allows for targeted biopsies and pancreatic fluid evaluation after lavage. POP may be used for IPMN visualization and targeted biopsy. A meta-analysis of 25 studies on different POP systems was performed, with the final evaluation based on surgical specimen evaluation, biopsy, or long-term follow-up observation. POP sensitivity was 64–100%, and POP specificity was 75–100% [125]. In a more recent multicenter cohort study, all patients underwent CT, MRI, or both prior to pancreatoscopy. Pancreatoscopy was performed due to MPD dilation and suspicion of MD-IPMN or MT-IPMN. At POP, different types of biopsies were taken, like brushing cytology of the pancreatic main duct (MD), fluid after irrigation of MD, or a forceps biopsy taken from the tumor. In 85% cases, POP may detect HDG, LGD, pancreatic stones, and mucus in MD. Based on the POP and biopsy, the decision was made for surgery, MRI follow-up, or pancreatic stenting. Among the 29 operated patients, 90% (26 patients) had correctly recognized pancreatic intraductal pathology. Most often, MD/MT-IPMN with LGD, MD/MT-IPMN with HGD, and MD/MT-IPMN with no dysplasia or no MD-IPMN but HDG in the irrigation fluid were detected [126]. In another single-center study of 36 patients, POP was performed due to a main papilla and MPD dilation. Imaging studies like CT, MRCP, or ultrasonography (USG) were performed prior to POP. A macroscopic view of the pancreatic duct was obtained, showing different IPMN morphologies. In the MPD protrusions, sessile, semipedunculated, villous, and vegetative lesions were determined. Similar lesions were detected in 2010 by Miura et al. [127]. During POP, cytology, biopsy, or both were performed on those protrusions. The specificity and sensitivity of biopsy/cytology obtained at POP were 85% and 87.5%. In the operated patients, different IPMN lesions showed various dysplasia grades. In seven sessile lesions, no dysplasia was determined. In ten, semipedunculated changes showed three adenocarcinomas and three atypical cells; three had no dysplasia, and one patient refused surgery. In villous lesions, eight adenocarcinomas and two atypical cells were found. In vegetative lesions, seven patients had adenocarcinoma, and two were followed up with an increased tendency to malignant transformation. The result shows that different IPMN morphologies are characterized by different dysplasia grades. Villous and vegetative lesions had the highest malignant potential and should be directed to surgical treatment. Semipedunculated lesions should be qualified for surgery based on cytological results. Cytology/biopsy under POP showed higher sensitivity and specificity values of 85% and 87.5% compared to EUS-FNA biopsy, with a sensitivity of 28.7% [37], making POP more accurate in diagnostic IPMN [128]. POP was performed in 27 IPMN patients with positive mural nodules. Twenty patients had WFs, and seven had HRS. Under POP, mural nodule biopsy and fluid sampling lavage were performed. With POP in 13 patients, definitive malignancy was diagnosed just after POP. Eight of them had malignancy determined in POP, and four had mural nodules >10 mm, which was the indication for immediate surgery. The remaining patient preferred surgical treatment despite a benign lesion detected in POP. In 9 of 13 patients who underwent operation just after POP, malignancy was confirmed with POP biopsy and pathology. The sensitivity and specificity of biopsy specimens obtained at POP were 63% and 100%, and fluid lavage in POP had sensitivity values of 89% and 100%. Twelve of thirteen specimens were confirmed with the final pathology, making POP-guided fluid ductal lavage more accurate than POP biopsy in detecting malignancy. Final pathology showed LG-IPMN in four patients, HG-IPMN in five patients, and invasive carcinoma in two patients, one of whom had PDAC and LG-IPMN, and one patient had mainly LG-IPMN with HG-IPMN [129]. The disadvantages of POP include the high cost, and special training is necessary. The pooled adverse events value from a meta-analysis of 17 studies was 12%. The most common one was post-endoscopic retrograde cholangiopancreatography (ERCP) pancreatitis, with a pooled rate of 10%. Overall, 70.6% had mild severity of post-ERCP pancreatitis, 20.6% had moderate, and 5.9% had severe. One death due to post-ERCP pancreatitis also occurred. Other adverse events were post-sphincterotomy bleeding at 1.3% and cholangitis at 8.3% The availability is also low. POP accuracy depends on MPD anatomy; ductal stenosis or possible pancreatic stones could be challenging, making this procedure not possible in some patients [125]. There are no available data on the comparison between POP and other diagnostic techniques. POP might be quite expensive due to the second scope (which also requires special maintenance), the second light source, and the special processor. POP probably is more expensive than CT or MRI [125,130].

All techniques are summarized in Table 9.

## 8. Conclusions

The proper diagnosis of IPMN, especially malignant lesions, despite improvements in technology, remains a significant diagnostic challenge. Currently, a combination of various techniques is used for this purpose, especially imaging, biomarker analysis, and histopathological examination. According to IAP 2024 and European 2018 Guidelines, MRI/MRCP is currently recommended as a prior diagnostic tool, while CT and EUS, with their additional techniques, should be introduced for further evaluation. AI-driven radiomics emerges as a new technology for IPMN diagnosis. It enables the application of deep learning processes to imaging data analysis and correlates these data with clinical information obtained from patients with the guidelines. Consequently, it boosts sensitivity and specificity in detecting malignancy in IPMN using MRI and CT. Cystic fluid analysis is another important tool. Glucose and CEA help determine whether a cyst is mucinous. Standard cytology shows a correlation with postsurgical pathology, with limited sensitivity but with good specificity. Among the advanced endoscopic procedures, EUS-nCLE offers in vivo histopathology, advancing immediate differentiation of malignant lesions. EUS-nCLE has high potential for routine use, but it is currently not widely available. Also in EUS-nCLE, AI-driven radiomics boosts sensitivity and specificity in determining malignant lesions, as well as correlates patients’ clinical data based on the guidelines. EUS-TTNB improves the collection of histopathology tissue samples and helps differentiate mucinous and non-mucinous lesions. New emerging markers, like mucin, miRNA, unique proteins, and metabolic changes, distinguish malignant lesions with high specificity and sensitivity and may be groundbreaking in the future. Lastly, pancreatoscopy remains valuable, especially in targeted biopsy of different IPMN lesions of high-grade dysplasia and cancer risk. In conclusion, traditional imaging and biomarker analysis remain the foundation of IPMN diagnosis. Future prospects, such as integrations of genetic diagnosis, AI-driven radiomics, and advanced endoscopic techniques, may improve early malignancy detection and risk stratification in IPMN. Further clinical studies are necessary to widen the use of these techniques in routine clinical practice.

## Figures and Tables

**Table 1:** Division of IPMN according to the origin.

BD-IPMN	Branch duct IPMN
MD-IPMN	Main duct IPMN
MT-IPMN	Mixed type IPMN

**Table 2:** Division of IPMN according to histological subtype.

Gastric
Intestinal
Pancreatobiliary
Oncocytic

**Table 3:** Division of IPMN according to the grade of dysplasia.

LG-IPMN	Low-grade IPMN
HG-IPMN	High-grade IPMN
IC-IPMN	Invasive carcinoma IPMN

**Table 4:** Comparison of the efficacy of commonly used techniques in IPMN diagnosis.

Technique	Sensitivity	Specificity	AUC	PPV	NPV	References
MRI	73.4–90.8%	64.4–94.8%	81.1–90.3%	71.00%	82.40%	[27,28,29,30,31]
MRCP	38.3–94.1%	62.5–93.1%	N/A	N/A	N/A	[27,31,32,33]
CT	62–90.4%	57–86%	71–90.4%	48–80%	58–90%	[29,30,31,34,35,36]
EUS	60%	80%	79%	N/A	N/A	[31]
EUS-FNA Cytology	28.7–64.8%	84–94%	84–94%	N/A	N/A	[37,38,39,40]
EUS-TTNB	69.50%	N/A	N/A	N/A	N/A	[37]

**Table 6:** Indications for surgery and surveillance in the case of IPMN according to different guidelines [7,21,25,46,47].

Guidelines	Indications for Surgery	Indications for Surveillance
AGA, 2015 [47]	Presence of a solid component, a dilated MPD, or concerning features on EUS or EUS-FNA.	Cyst < 3 cm without solid component or dilated MPD should undergo MRI surveillance in 1 year and then every 2 years and a total of 5 years if there is no change in size or characteristics.
ACR, 2017 [46]	Presence of HRS or WFs, according to ACR, should prompt EUS-FNA and surgery. **HRS**: obstructive jaundice with a cyst in the head of pancreas, enhancing solid component within a cyst, MPD caliber ≥ 10 mm in the absence of obstruction. **WFs**: cyst ≥ 3 cm, thickened/enhancing cyst wall, non-enhancing mural nodule, MPD caliber ≥ 7 mm.	Cyst > 3 cm without any additional HRS or WFs; 9–10 years follow-up based on initial size.
European, 2018 [25]	**Absolute**: jaundice, enhancing mural nodule > 5 mm or solid component, MPD diameter > 10 mm, positive cytology. **Relative**: MPD diameter 5–9.9 mm, cyst diameter > 40 mm, cyst growth rate > 5 mm/year, symptoms, enhancing mural nodules < 5 mm, new onset of DM, acute pancreatitis, CA 19-9 > 37 U/mL.	Patients without absolute or relative indications for surgery should be placed under surveillance. A 6-month follow-up in the first year, then yearly follow-ups are recommended. For patients with relative indications for surgery, the “elderly”, and those affected by severe comorbidity, a 6-month follow-up is recommended.
ACG, 2018 [7]	Any of the following symptoms: jaundice, acute pancreatitis, and elevated serum level CA 19-9. Any of the following imaging findings: presence of mural nodule or solid component, dilation of MPD > 5 mm, focal dilation of the MPD, cyst diameter ≥ 3 cm, and positive cytology. The decision whether or not to resect a cystic lesion is best determined by a pancreatic team that integrates multiple different factors, such as patient comorbidities and life expectancy.	Pancreatic cyst surveillance should be offered to surgically fit patients with asymptomatic IPMNs or MCNs. The cyst surveillance strategy is stratified based on cyst size. Cyst < 1 cm: MRI every 2 years for 4 years. Cyst 1–2 cm: MRI every 1 year for 3 years. Cyst 2–3 cm: MRI or EUS every 6–12 months for 3 years. Cyst > 3 cm: MRI or EUS every 6 months for 3 years, consider referral to multidisciplinary group. Surveillance should be stopped after 5 years if there are no high-risk features and the size of the cyst is stable.
IAP, 2024 [21]	Presence of any HRS or WF. **HRS**: obstructive jaundice in a patient with a cystic lesion of the head of the pancreas, enhancing mural nodule ≥ 5 mm or solid component, MPD ≥ 10 mm, suspicious or positive results of cytology. **WFs**: acute pancreatitis, increased serum level of CA 19-9, new onset or acute exacerbation of DM within the past year, cyst ≥ 30 mm, enhancing mural nodule < 5 mm, thickened/enhancing cyst walls, MPD ≥ 5 mm and <10 mm, abrupt change in caliber of the pancreatic duct with distal pancreatic atrophy, lymphadenopathy, cystic growth rate ≥ 2.5 mm/year.	Absence of HRS and WFs. Surveillance scheme depends on initial cyst size: Cyst < 20 mm: 6 months once, then every 18 months. Cyst 20–30 mm: 6 months twice, then every 12 months. Cyst > 30 mm: every 6 months. Surveillance may be stopped after 5 years, if the cyst is stable.

**Abbreviations:** IAP, International Association of Pancreatology; ACG, American College of Gastroenterology; ACR, American College of Radiology; AGA, American Gastroenterological Association.

**Table 7:** CT role in malignancy detection in IPMN.

Technique	Additional Features Assessed	Sensitivity	Specificity	Accuracy	PPV	NPV	AUC	References
CT	HRS, WFs (2017 ICG Fukuoka)	79.5–86%	67.8–74%	73.7–78%	71.40%	76.60%	-	[29,30]
CT + radiomics	-	68–82%	57–84%	64–78%	48–80%	58–90%	0.71–0.84	[34,35,36]
	HRS, WFs (2017 ICG Fukuoka, 2018 European)	69–80%	65–72%	67–76%	72–78%	61–75%	0.75–0.83	[34]
	Age, cyst size, presence of solid component, symptoms, gender	19–93%	35–100%	50–80%	36–97%	78–95%	0.74–0.81	[35,61]
	Type of IPMN, cyst size, cystic solid, CA199, CA125, bilirubin, alkaline phosphase, gamma-ggtt, diabetes	90.40%	74%	80%	-	-	0.904	[36]

**Table 8:** The role of EUS-nCLE in malignancy detection in IPMN.

Type of Different Detection PCLs	Sensitivity	Specificity	AUC	Accuracy	References
Detection of HGD or IC in IPMN	87–90%	73–100%	0.95	83%	[105]
Detection of HGD or IC in BD-IPMN by humans without revised Fukuoka HRS and WFs	58.20%	58.80%	0.59	58.50%	[106]
Detection of HGD or IC in BD-IPMN by humans with revised Fukuoka HRS and WFs	72%	56.80%	0.64	62.60%	
Detection of HGD or IC in BD-IPMN by AI-algorithm without revised Fukuoka HRS and WFs	87%	54.10%	0.7	66.70%	
Detection of HGD or IC in BD-IPMN by an AI algorithm with revised Fukuoka HRS and WFs	78.30%	78.40%	0.85	78.30%	
Differentiating mucinous from non-mucinous lesions	95.20%	94.20%	N/A	94.80%	[107]
Differentiating IPMN from other PCLs	84.40%	88%	N/A	86.20%	

**Table 9:** Comparison of the advantages and disadvantages of the techniques described in this review.

Technique	Advantages	Disadvantages
MRI	Effective in detection mural nodules > 5 mm Detecting PDFF No radiation	Less effective in detecting MPD dilation Relatively long-lasting examination Less available than CT
CT	Shorter examination than MRI More available than MRI	Radiation Lower image resolution
Radiomics—MRI and CT	Analysis of quantitative features normally not available for humans AI algorithm integrates image features with clinical data	Still in research phase, not yet available in clinical practice
EUS	High resolution in detecting mural nodules and vascularity visualization Enables acquisition of cyst fluid and cytology No radiation	Training required High dependence on experience of the operators
EUS-FNA, cyst fluid analysis	Routinely assessed markers useful for differentiating mucinous and non-mucinous cysts	Routinely assessed markers ineffective in differentiating malignancy Complications: pancreatitis, pain, infection Training required
EUS-FNA, cytology	Highly specific in detecting malignancy	Insufficient sensitivity in detecting malignancy
EUS-TTNB	Enabling acquisition of macroscopically visible tissue samples Differentiating mucinous from non-mucinous as well as malignant cysts	Training required Complications: bleeding, pancreatitis, infections
EUS-FNB	Better preserved tissue architecture	Training required Complications similar to EUS-FNA
EUS-nCLE	Enables in vivo imaging similar to histopathology Detection of malignancy in IPMN as well as mucinous cysts	High cost Training required Not widely available Complications: pancreatitis, bleeding, abdominal pain
New markers: mucins, miRNA, metabolic profile, Das-1 antibody	Biomarkers present strong association with malignancy risk of IPMN, available to assess from cyst fluid or blood	Not widely available High cost
Pancreatoscopy	Enables direct visualization of MPD Effective in the detection malignancy of IPMN Biopsy under POP more efficient than EUS-FNA biopsy	Training required Complications: more common pancreatitis than EUS-TTNB and EUS-nCLE High cost Not widely available
